# Procedures in pediatric primary care: first do no harm

**DOI:** 10.1186/s13584-015-0051-6

**Published:** 2015-12-10

**Authors:** Gary L. Freed

**Affiliations:** Child Health Evaluation and Research Unit, University of Michigan, 300 North Ingalls, Ann Arbor, MI 48109 USA; University of Melbourne, Parkville, 31 61 Victoria Australia

## Abstract

Two important considerations arise from this IJHPR article from Zimmerman and colleagues. First, is the question regarding what can be considered a “common” procedure in primary care and whether the designation can or should change over time. The second issue is whether it is enough for a doctor to feel comfortable doing a procedure for it to be within their scope of practice, or whether the practice specific outcome for the procedure in terms of safety and efficacy is a more relevant determination of whether the procedure should be performed in a given setting. In other words, just because a doctor “can” or “wants” to do a procedure, may not mean they “should” do a procedure.

The role of procedures in a practice of primary care also differs markedly in the care of children vs. the care of adults. This phenomenon is partially the result of the more challenging aspects of the care of infants and small children with regard to the ability to maintain a sterile field for procedures, and the relative infrequency with which procedures are performed on children relative to adults.

The scope of practice for pediatricians in the community has changed over time and is likely to continue to change. This paper helps to define the current state of practice for paediatricians with regard to the conduct of 10 specific procedures. It challenges us to think about the appropriateness of the venue of care and its implications for both the status quo and the future of community based primary care.

## Background

This provocative article by Zimmerman et al. [1] raises a number of important issues regarding the scope of care provided by primary care doctors in general, and by pediatric primary care physicians specifically. The question of which procedures can or should be performed in outpatient settings has many different perspectives. The issue may also viewed in the context of what procedures are “appropriate” for a primary care doctor to perform.

Zimmerman and colleagues raise numerous issues that may influence the decision of a primary care paediatrician to refer a patient for a procedure. These include the training programs of doctors during residency, the environment in which they practice, their recent experience with the procedure, a perceived lack of time or payment, and failure of doctors to stay “up to date” with the specific skills required. The authors examine how frequently doctors refer patients for 10 “common” procedures and why they make the decision to refer.

### Defining a “common” procedure

Two important considerations arise from this work. First, is the question regarding what can be considered a “common” procedure in primary care and whether the designation can or should change over time. The second issue is whether it is enough for a doctor to feel comfortable doing a procedure for it to be within their scope of practice, or whether the practice specific outcome for the procedure in terms of safety and efficacy is a more relevant determination of whether the procedure should be performed in a given setting. In other words, just because a doctor “can” or “wants” to do a procedure, may not mean they “should” do a procedure.

When determining what is a “common” procedure, the paper’s own findings are illustrative of the difficulty in establishing that label. Four of the 10 procedures examined in this paper are routinely performed by fewer than 50 % of those participating in the study, and two are performed by fewer than 30 %. Indeed, is the performance of these procedures now the exception or the rule? If so, can they still be termed “common” in primary care and by whose definition? If not, is that necessarily a bad thing?

Although this may seem semantic, it cuts to the issue of what the public may expect of a pediatrician in 2015, relative to what may have been expected in years past. Many clinicians (myself included) often lament when things change and bemoan those changes. For most outpatient procedures in primary care, we only have anecdotal reports of what proportion of doctors actually performed them at any given time in the recent or distant past. I hypothesize that those who performed procedures were much more likely to talk about them than those who did not, perhaps leading to skewed perception. In fact some procedures which (by anecdote) were commonly performed in the outpatient setting in years past, may not have been performed as commonly as we now opine.

In the United States, in the 1950s, and into the 1960s and 1970s there were procedures performed in primary care that would be unthinkable today. Some paediatricians and family physicians performed tonsillectomies and “rolled their own plaster” for fractures in their private clinics. Such procedures were not performed only in rural communities, but also in suburban practices. The outcomes of such procedures overall are unknown. However, liability concerns and the potential for bad outcomes likely had a role the performance of these procedures in the primary care setting over the past few decades. Parental perceptions and expectations may also have played a role.

### The role of patient safety and quality of care

The other, more important, issue is in which venue is it in the best interest of the patient for a specific procedure to be performed. Just because a primary care pediatrician enjoys performing a procedure in the outpatient setting does not mean it is necessarily in the best interest of the patient for him or her to do so.

As the emphasis on quality of patient care has gained traction, the debate over by whom, and where, procedures should be performed has taken on a new context. The medical literature is replete with data that, in general, demonstrate those who perform procedures more commonly have better outcomes for those procedures [2, 3]. Thus, although being trained to perform a procedure, and having competence at one point in one’s career is important, it does not confer lifelong proficiency.

As such, the findings of Zimmerman et al. regarding the more common performance of specific procedures in the years shortly following training, compared with later years, are not surprising. Once in a community based practice, the frequency with which procedures are performed likely drops off. Some doctors may also be hesitant to perform procedures in a less controlled setting as well. For example, it appears in this study that those procedures requiring a sterile field were among those most likely to be referred (e.g., suture of laceration, supra-pubic aspiration).

With specific regard to quality, the complication rate for specific procedures performed in a community setting is unknown nor are there any quality measures in this arena that have been tested for reliability and validity. Further, although the authors rightfully point out that lacerations are a common occurrence in childhood, the frequency with which one must perform suturing to maintain competence is also unknown. As such, although there are specific benefits to patients for some procedures to be performed in a community setting, there may be differences in the quality of the procedure performed. Until there are better data regarding outcomes and complications to compare the conduct of specific procedures in different settings, or a set of defined quality metrics, it is difficult to assume referral is a less desirable option for some procedures.

### Utility of specific procedures

Finally, the procedure in this study with the highest rate of referral was the clipping of a short frenulum. This uncommonly performed procedure is most frequently used to address breastfeeding latch-on problems. However, it may not be the best example of the issue of referral. The condition only occurs in 0.2 % of infants and most instances are not pathologic or actually interfere with breastfeeding. It is unknown in what proportion of cases the procedure actually improves breastfeeding [4]. Additionally, most cases recede in the first year of life without intervention. It is also unclear from this study whether the decision to refer this condition also includes the desire for a pediatrician to determine if the procedure is actually required.

The role of procedures in a practice of primary care also differs markedly in the care of children vs. the care of adults. It is not surprising that the references provided by Zimmerman, et al. on this issue are exclusively regarding procedures performed on adults. This phenomenon is partially the result of the more challenging aspects of the care of infants and small children with regard to the ability to maintain a sterile field for procedures, and the relative infrequency with which procedures are performed on children relative to adults.

## Conclusions

The scope of practice for pediatricians in the community has changed over time and is likely to continue to change. This paper helps to define the current state of practice for paediatricians with regard to the conduct of 10 specific procedures. It challenges us to think about the appropriateness of the venue of care and its implications for both the status quo and the future of community based primary care. Future studies that can help to shed light on any differences in the quality of care and patient outcomes depending on where procedures are performed will help to guide both future training requirements and scope of practice.
